# Occipital Extracranial Dermoid Cyst in a Neonate With Cardiofaciocutaneous Syndrome Type 4 (CFC4): A Case Report

**DOI:** 10.1002/ccr3.71752

**Published:** 2026-01-08

**Authors:** Mona Alkallabi, Khalid Nabil Nagshabandi, Naif Ahmed Alshehri, Hala Abdullah Almusa, Almunthir Saud Alhamed

**Affiliations:** ^1^ Department of Dermatology College of Medicine, King Saud University Riyadh Saudi Arabia; ^2^ Department of Dermatology and Allergy, Clinical Research Center for Hair and Skin Science Charité‐ Universitätsmedizin Berlin Germany

**Keywords:** cardiofaciocutaneous syndrome type 4, dermoid cyst, epidermoid cyst, genetic syndrome, MAP2K2 mutation, occipital scalp, RASopathies

## Abstract

Dermoid cysts are congenital inclusion lesions that arise from ectodermal entrapment along embryonic fusion lines; occipital extracranial involvement is particularly uncommon. Cardiofaciocutaneous syndrome type 4 (CFC4), a RASopathy caused by pathogenic variants in MAP2K2, presents with characteristic dermatologic, craniofacial, and multisystem findings. We report an occipital extracranial dermoid cyst in a neonate with CFC4, proposing a possible developmental association between these two rare entities. A 16‐day‐old female infant, born preterm at 28 weeks, presented with multiple firm, skin‐colored nodules on the occipital scalp. Physical examination revealed three discrete nodules with overlying alopecia and variable fixation to deeper structures. The initial differential diagnosis included dermoid cyst, epidermoid cyst, osteoma cutis, and calcinosis cutis. Neuroimaging demonstrated well‐defined extracranial soft tissue masses without intracranial extension, supporting the diagnosis of an occipital dermoid cyst. Genetic testing was pursued due to dysmorphic features and confirmed a pathogenic MAP2K2 mutation, consistent with CFC4. In the absence of mass effect, ulceration, or neurologic compromise, conservative management and close clinical follow‐up were recommended. Serial evaluations showed stability without progression. This case highlights an atypical occipital extracranial dermoid cyst in the setting of CFC4 and emphasizes two practical points: first, atypical scalp lesions in neonates warrant early imaging to exclude intracranial connection; second, syndromic evaluation (including molecular testing) should be considered when such lesions coexist with dysmorphic or ectodermal findings. Further observation and accumulation of similar cases are needed to explore a potential link between RASopathies and abnormal ectodermal/developmental fusion along the posterior scalp.

## Key Clinical Message

This case highlights the first reported association between an occipital dermoid cyst and autosomal dominant cardiofaciocutaneous syndrome type 4, emphasizing the importance of genetic evaluation and imaging in neonates with atypical scalp lesions and syndromic features.

## 1 Introduction

Dermoid cysts are benign lesions lined by squamous epithelium that congenitally arise as a result of failure of the ectoderm to separate from the neural tube [1]. They may occur anywhere in the body, but are most commonly reported in the ovary and scrotal regions. Only about 7% are found in the head and neck region. Within the head and neck, typical sites include the lateral eyebrow and the floor of the mouth, reflecting embryonic fusion lines [2]. Intracranial dermoids are rare and are sometimes associated with overlying cutaneous scalp lesions [3]. Occipital extracranial dermoid cysts in neonates are particularly uncommon [4, 5].

This case study highlights the clinical presentation, diagnostic workup, and management options of an occipital cranial dermoid cyst in a patient genetically confirmed to have autosomal dominant cardiofaciocutaneous syndrome type 4 (CFC4), emphasizing its rarity and the anatomical nuances associated with this uncommon site.

## 2 Case History/Examination

**FIGURE 1:**
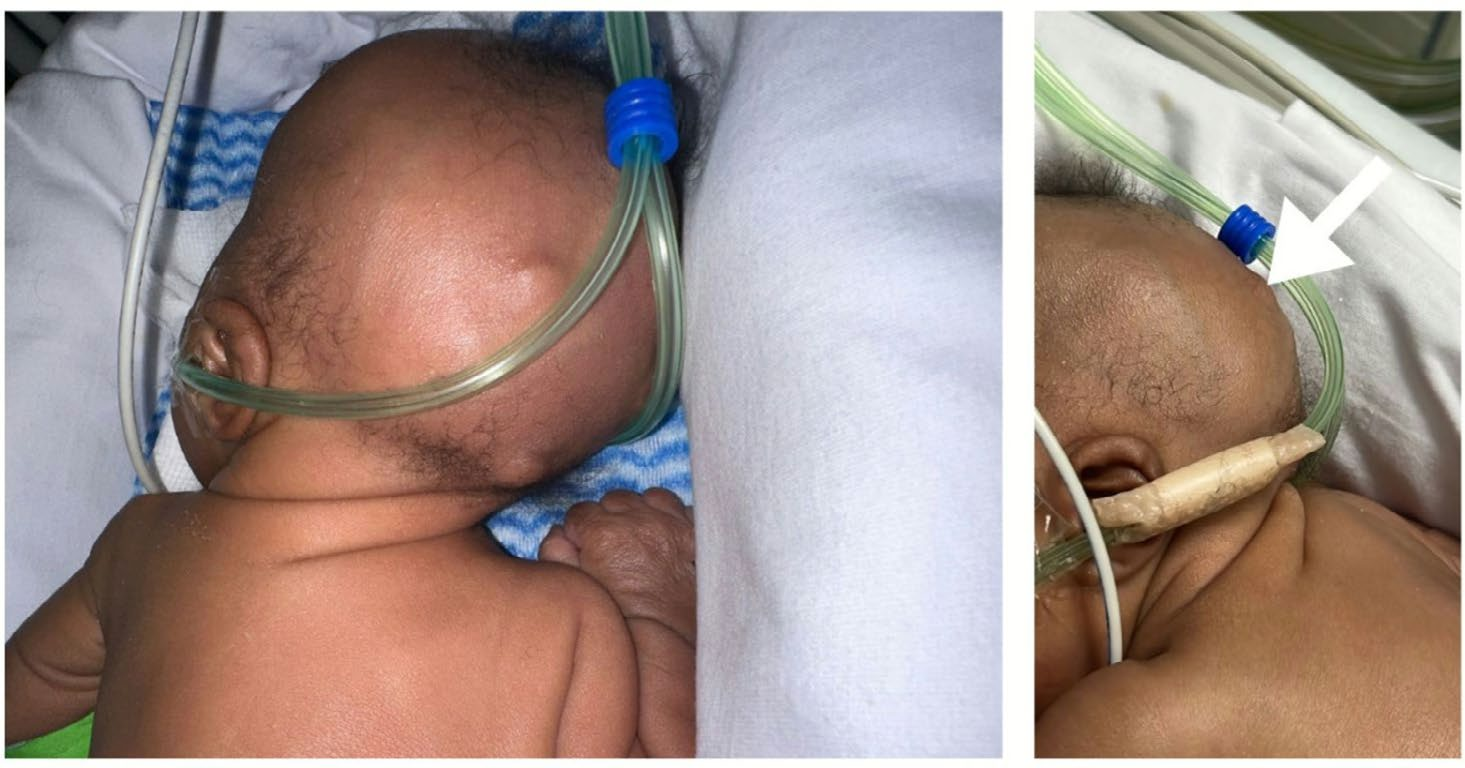
Clinical photographs of an infant with three firm, deep‐seated nodules on the occipital scalp (white arrow).

A 16‐day‐old female infant, born at 28 weeks gestation and weighing 1.78 kg, was first evaluated by the dermatology team with a chief complaint of nodules over the occiput, noticed 1 week prior. The infant had a complex neonatal course, with a history of respiratory distress syndrome, hydronephrosis, hypocalcemia, large patent ductus arteriosus, and bilateral congenital cataract. The patient also exhibited dysmorphic features, raising concerns about a possible underlying genetic syndrome. On physical examination, three discrete firm, skin‐colored, deep‐seated nodules were identified on the occipital scalp, with overlying alopecia. The inferior nodule was firm and fixed to deeper structures, with superficial microerosions, while the two superior nodules were erythematous, rubbery in texture, and mobile. The skin overlying all nodules was freely movable, and none of the lesions showed signs of discharge or ulceration. No other lesions were noticed on the body (Figure 1). There was no known family history of similar lesions or genetic syndromes.

## 3 Differential Diagnosis, Investigations and Treatment

**FIGURE 2:**
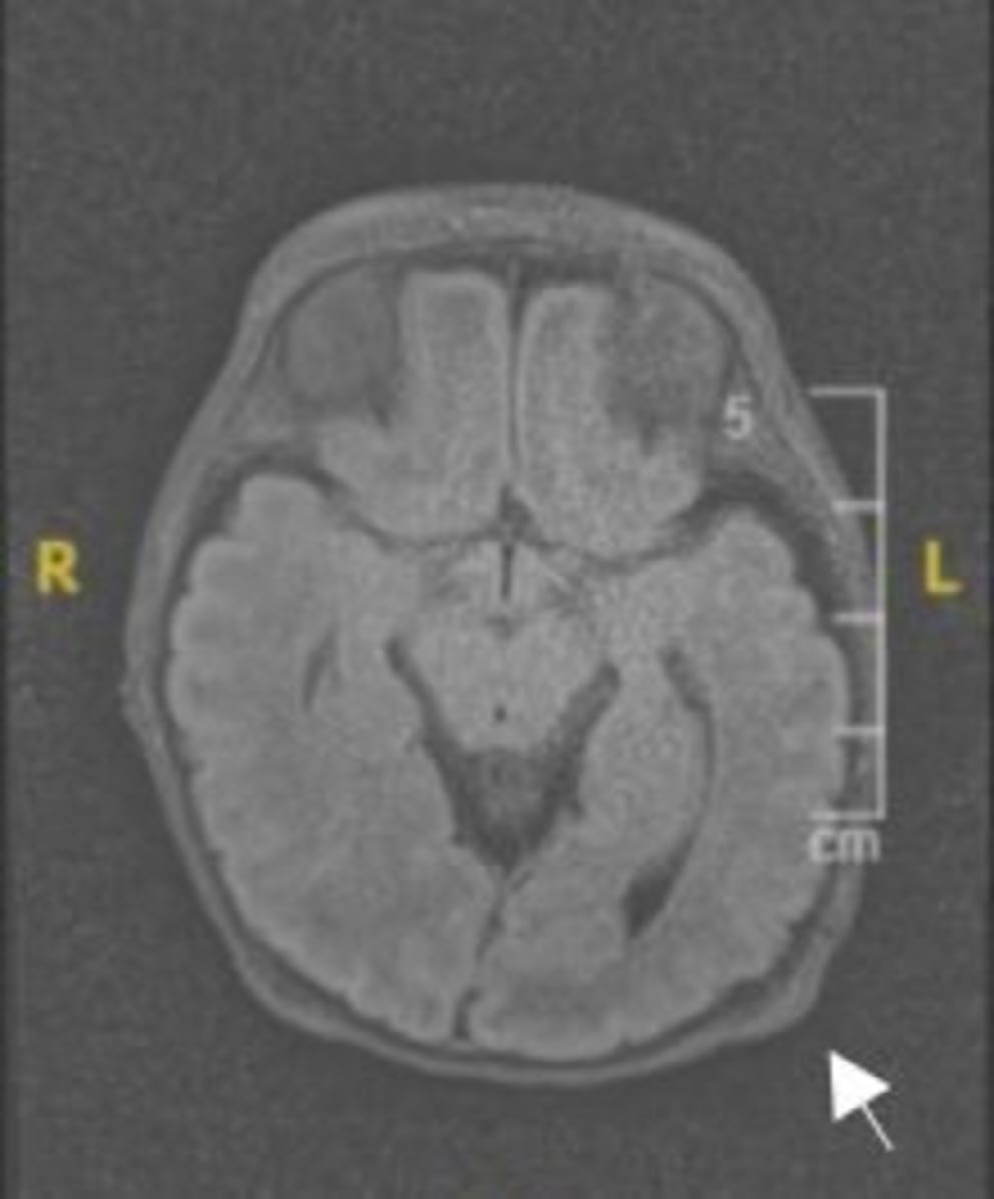
Axial T2‐weighted MRI of the occipital region in a preterm neonate. White arrow indicate well‐defined extracranial soft tissue nodules along the occipital scalp. There is no intracranial extension.

Given the presentation, initial differential diagnoses included dermoid cyst, epidermoid cyst, osteoma cutis, and calcinosis cutis. An MRI was recommended by the team to further evaluate the lesion and to rule out any deeper extension; however, it was not initially completed. At the second visit (day 30 of life), MRI subsequently demonstrated a well‐defined occipital extracranial mass with no intracranial extension or involvement of deeper structures (Figure 2). Genetic testing was performed at 75 days of life due to the patient's dysmorphic features and other congenital anomalies. Whole exome sequencing (WES) identified a heterozygous pathogenic variant in the MAP2K2 gene (c.181A>G p.(Lys61Glu)), confirming the diagnosis of autosomal dominant cardiofaciocutaneous syndrome type 4 (CFC4).

Ultimately, the diagnosis of an occipital dermoid cyst was established clinically and supported by imaging findings. Following the diagnosis, a conservative management approach was recommended. The neurosurgery team was consulted, and given the absence of intracranial connection, they determined that surgical intervention was not necessary.

## 4 Conclusion and Results (Outcome and Follow‐Up)

**FIGURE 3:**
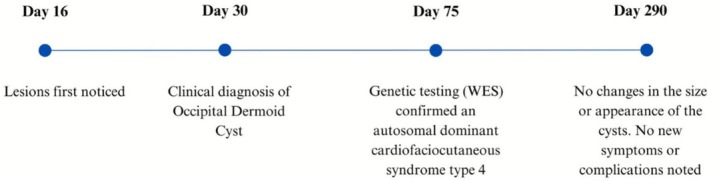
Case timeline of events.

At the most recent follow‐up, the occipital nodules remained stable in size and appearance without complications, and no new symptoms developed. Given the benign nature of the lesion and the lack of progression, there was no need for confirmatory biopsy. Surgical excision may be considered for cosmetic reasons or if complications arise, such as infection or rapid growth. Regular monitoring for changes in the lesion will also be important as the child grows. Figure 3 provides a timeline summarizing the key events in the patient's clinical course. All procedures performed in this study were in accordance with the ethical standards of the institutional and/or national research committee(s) and with the Helsinki Declaration (as revised in 2013). Written informed consent was obtained from the patient parents for publication of this case report and accompanying images.

## 5 Discussion

Dermoid cysts are rare, benign congenital lesions arising from the entrapment of ectodermal elements during neural tube closure. They commonly contain sebaceous glands, hair follicles, and sweat glands, encapsulated by squamous epithelium [1]. They characteristically develop along embryonic fusion lines, including sites in the head and neck and the sacrococcygeal region [5, 6]. Approximately 7% occur in the head and neck, with the lateral eyebrow and the floor of the mouth being frequent sites. Intracranial dermoid cysts are less common, accounting for 0.04%–0.7% of intracranial tumors [6, 7]. The pathogenesis involves ectodermal remnants becoming trapped during the neural tube's midline closure. These remnants differentiate into skin structures and form cystic masses that may coexist with other congenital anomalies. In rare cases, these cysts may rupture, releasing irritants into the surrounding tissues and leading to aseptic meningitis or other complications [1, 5].

Occipital scalp dermoid cysts are distinctly uncommon and require careful assessment because of their proximity to critical neurovascular structures and the possibility of intracranial extension [8]. Posteriorly located dermoid cysts have been associated with genetic and developmental disorders such as Klippel–Feil syndrome (KFS) and tethered cord syndrome, which involve segmentation anomalies of the axial skeleton [7, 9, 10, 11]. Reports of posterior scalp involvement in the context of a defined syndromic diagnosis remain limited. Our case adds a possible association with autosomal dominant cardiofaciocutaneous syndrome type 4 (CFC4), expanding the discussion of how RASopathies may intersect with atypical dermoid locations.

Cardiofaciocutaneous syndrome (CFC) is a rare RASopathy caused by dysregulation of the RAS–MAPK signaling pathway. It overlaps clinically with Noonan and Costello syndromes and is characterized by craniofacial dysmorphism, cardiac defects, neurologic/developmental delay, and characteristic cutaneous findings. CFC type 4 (CFC4; OMIM #615278) is associated with pathogenic variants in MAP2K2 and is typically inherited in an autosomal dominant fashion, most often arising de novo [12]. Cutaneous features include xerosis, keratosis pilaris (also historically described as “follicular hyperkeratosis”), eczema‐like lesions, ichthyosis, café‐au‐lait macules, and sparse, brittle, slow‐growing hair [12]. In this context, the coexistence of characteristic ectodermal changes and an atypically located scalp mass supports the need to consider an underlying syndromic diagnosis rather than viewing the lesion in isolation.

Surgical excision remains the mainstay of management for scalp dermoid and epidermoid cysts. Preoperative imaging with CT or MRI is critical to evaluate the lesion's extent, bony involvement, and proximity to key structures, and to determine whether there is any intracranial communication. An important diagnostic consideration in this setting is the distinction between a dermoid cyst and an epidermoid cyst. Dermoid cysts typically contain adnexal structures (hair follicles, sebaceous elements), which produce lipid‐rich internal material that can appear hyperintense on T1‐weighted MRI. Epidermoid cysts, by contrast, are filled with keratinaceous debris and often demonstrate diffusion restriction on MRI. However, dermoid cysts can rarely mimic the signal behavior of epidermoid cysts, including features on diffusion‐weighted imaging, which can complicate preoperative interpretation. This overlap has been described in recent neuroradiologic literature and underscores why correlation with location, clinical stability, and absence of intracranial extension was crucial in our case [13]. When rupture, infection, or abscess formation occurs, additional measures such as antibiotics or drainage may be needed [1, 2, 8]. In stable extracranial lesions without intracranial extension or functional compromise, observation can be reasonable in early infancy while growth, neurologic status, and overlying skin are monitored. Interdisciplinary input (dermatology, neurosurgery, radiology, genetics) is especially important in atypical locations.

In the present case, a neonate presented with multiple firm nodules on the occipital scalp, prompting a differential that included dermoid cyst, epidermoid cyst, osteoma cutis, and calcinosis cutis. MRI demonstrated well‐defined extracranial masses without intracranial extension, which supported conservative rather than immediate surgical management. Whole exome sequencing subsequently identified a pathogenic MAP2K2 variant, confirming CFC4. This sequence of evaluation highlights two practical priorities in similar neonates: first, early imaging of atypical scalp nodules to exclude deeper extension; second, syndromic/genetic assessment when such nodules coexist with dysmorphic features or other congenital anomalies. Avoiding reflex early excision in a clinically stable neonate also minimizes procedural risk until neurosurgical intervention, if ever needed, can be more safely timed.

Limitations of this report include the lack of histopathological confirmation, which remains the diagnostic gold standard for dermoid cysts. Additionally, the absence of long‐term follow‐up data limits assessment of potential progression, such as interval enlargement or secondary infection. Another limitation is that the proposed association between an occipital extracranial dermoid cyst and CFC4 is based on a single observation, so a causal link cannot be inferred. Future work, including additional case reports and small series with genetic characterization, will be needed to clarify whether RASopathies predispose to atypical dermoid locations.

Despite these limitations, this case contributes several points. First, it documents an occipital extracranial dermoid cyst without intracranial extension in a neonate with confirmed CFC4. Second, it outlines a conservative management pathway supported by targeted imaging and neurosurgical input. Third, it argues for incorporating genetic testing into the evaluation of neonates with atypical congenital scalp nodules and dysmorphic features, rather than treating each finding in isolation.

## 6 Conclusion

Dermoid cysts are uncommon congenital lesions that can pose diagnostic and management challenges, particularly when they arise in atypical posterior scalp locations. This case describes an occipital extracranial dermoid cyst in a neonate with CFC4 and emphasizes that atypical scalp lesions in early life should prompt both timely imaging to rule out intracranial extension and consideration of an underlying syndromic diagnosis. This observation supports a multidisciplinary approach and raises a hypothesis that CFC4 may present with unusual dermoid cyst locations.

## Author Contributions

**Mona Alkallabi:** conceptualization, project administration, supervision, validation, writing – review and editing. **Khalid Nabil Nagshabandi:** conceptualization, data curation, investigation, methodology, validation, writing – original draft, writing – review and editing. **Naif Ahmed Alshehri:** methodology, visualization, writing – original draft, writing – review and editing. **Hala Abdullah Almusa:** methodology, visualization, writing – original draft, writing – review and editing. **Almunthir Saud Alhamed:** data curation, investigation, methodology, project administration, resources, writing – original draft, writing – review and editing.

## Funding

The authors have nothing to report.

## Ethics Statement

The authors are accountable for all aspects of the work in ensuring that questions related to the accuracy or integrity of any part of the work are appropriately investigated and resolved. All procedures performed in this study were in accordance with the ethical standards of the institutional and/or national research committee(s) and with the Helsinki Declaration (as revised in 2013). Written informed consent was obtained from the patient parents for the publication of this case report and accompanying images.

## Conflicts of Interest

The authors declare no conflicts of interest.

## Data Availability

The data that support the findings of this study are available from the corresponding author upon reasonable request.
